# Characterization and modulation of microglial phenotypes in an animal model of severe sepsis

**DOI:** 10.1111/jcmm.14606

**Published:** 2019-10-26

**Authors:** Monique Michels, Mariane Rocha Abatti, Pricila Ávila, Andriele Vieira, Heloisa Borges, Celso Carvalho Junior, Diogo Wendhausen, Juciano Gasparotto, Camila Tiefensee Ribeiro, José Claudio Fonseca Moreira, Daniel Pens Gelain, Felipe Dal‐Pizzol

**Affiliations:** ^1^ Laboratory of Experimental Pathophysiology, Graduate Program in Health Sciences University of Southern Santa Catarina Criciúma SC Brazil; ^2^ Centro de Estudos em Estresse Oxidativo, Departamento de Bioquímica Instituto de Ciências Básicas da Saúde, Universidade Federal do Rio Grande do Sul Porto Alegre RS Brazil; ^3^ Departamento de Civil y Ambiental Universidad de la Costa Barranquilla Atlántico Colombia

**Keywords:** inflammation, M1/M2, Microglia, microglial polarization, phenotypes, sepsis

## Abstract

We aim to characterize the kinetics of early and late microglial phenotypes after systemic inflammation in an animal model of severe sepsis and the effects of minocycline on these phenotypes. Rats were subjected to CLP, and some animals were treated with minocycline (10 ug/kg) by i.c.v. administration. Animals were killed 24 hours, 5, 10 and 30 days after sepsis induction, and serum and hippocampus were collected for subsequent analyses. Real‐time PCR was performed for M1 and M2 markers. TNF‐α, IL‐1β, IL‐6, IL‐10, CCL‐22 and nitrite/nitrate levels were measured. Immunofluorescence for IBA‐1, CD11b and arginase was also performed. We demonstrated that early after sepsis, there was a preponderant up‐regulation of M1 markers, and this was not switched to M2 phenotype markers later on. We found that up‐regulation of both M1 and M2 markers co‐existed up to 30 days after sepsis induction. In addition, minocycline induced a down‐regulation, predominantly, of M1 markers. Our results suggest early activation of M1 microglia that is followed by an overlap of both M1 and M2 phenotypes and that the beneficial effects of minocycline on sepsis‐associated brain dysfunction may be related to its effects predominantly on the M1 phenotype.

## 1 INTRODUCTION

Sepsis is characterized by excessive production of inflammatory mediators resulting in deregulated host response and organ dysfunction that includes brain dysfunction. In this context, many studies have already reported microglia activation in animal models and humans.^1^, ^2^, ^3^, ^4^, ^5^, ^6^, ^7^ The primary role of microglia is the maintenance of neural cells and synapses during normal functioning of the CNS. Upon harmful stimulus, microglia are activated and can have a protective role. Activated microglia are very plastic and may exist in multiple phenotypes that present different responses in accordance to changes in the cerebral microenvironment. They can induce brain repair, or cytotoxicity, as well as immune‐regulatory or pro‐inflammatory functions.^8^, ^9^

Classically, microglial and macrophage polarization into M1 and M2 phenotypes is often discussed using similar terminology although this dichotomous division is oversimplified.^10^ Theoretically, these different phenotypes can have negative or positive effects on adult brain adaptation after damage. When activated as M1 phenotype, microglia affect neurogenesis,^11^, ^12^ secrete pro‐inflammatory mediators (CD11b, iNOS, CD32, nitric oxide) and worsen long‐term neurological deficits after damage.^13^ On the other hand, when activated as M2 phenotype, they promote repair, regeneration and neuroprotection through markers Arg‐1, CCL‐22, IL‐10, CD206 and TGF.^12^, ^14^ Minocycline has been reported to suppress the production of inflammatory mediators (IL‐6, TNF‐*α* and IL‐1*β*) induced by LPS in peripheral cells^15^ and has the same effect when injected into the brain,^3^ suggesting an effect mainly on M1 microglia.

The kinetics of microglial polarization during sepsis development is not known, and it can have implications in the recovery of brain function. Furthermore, the effect of minocycline on microglial phenotypes in sepsis is also unknown. Thus, we aimed to characterize the kinetics of microglial phenotypes early and late after systemic inflammation in an animal model of severe sepsis, and the effects of minocycline on these phenotypes.

## 2 MATERIALS AND METHODS

### 2.1 Ethics

The experimental procedures involving animals were performed in accordance with the National Institutes of Health (Bethesda—Maryland, USA) Guide for Care and Use of Laboratory Animals and with the approval of our institutional ethics committee (protocol number: 084‐2015/1). Animals were allocated and randomized as five animals per cage. Only one person knew the groups; all others were blinded in surgery for CLP (caecal ligation and perforation) and analyses.

### 2.2 Sepsis induction—CLP model

Male *Wistar r*ats were subjected to CLP as previously described.^16^ Briefly, animals were anaesthetized using a mixture of ketamine (80 mg/kg) and xylazine (10 mg/kg), administered intraperitoneally. Under aseptic conditions, a 3 cm midline laparotomy was performed to expose the caecum and adjoining intestine. The caecum was ligated with a 3.0 silk suture at its base, below the ileocaecal valve, and was perforated once with a 14‐gauge needle. The caecum was then squeezed gently to extrude a small amount of faeces through the perforation site. The caecum was then returned to the peritoneal cavity, and the laparotomy was closed with 4.0 silk sutures. Animals were immediately resuscitated with saline (50 mL/kg) subcutaneously (s.c.) immediately and 12 h after CLP. All animals received antibiotics (ceftriaxone at 30 mg/kg and clindamycin 25 mg/kg) every 6 hours s.c. for a maximum of 3 days. To minimize variability between different experiments, the CLP procedure was always performed by the same investigator. The mortality rate of this model is around 40%, which is consistent with severe sepsis. We extensively characterized acute and long‐term cognitive impairment and brain inflammation using this animal model.^3^, ^17^, ^18^, ^19^

### 2.3 Experimental design

Animals were subjected to CLP, and one group was treated with a single intracerebroventricular (i.c.v.) injection of minocycline (100 µg/kg) immediately after sepsis induction.^3^ Using this treatment regime, we had demonstrated that minocycline was able to decrease brain inflammation, damage and long‐term cognitive deficits in sepsis survivors.^3^ Animals were killed immediately after CLP (time 0), 24 hours, 3, 5, 10 and 30 days after sepsis, and the hippocampus and serum were removed and stored at −80°C. For all analyses, CLP time 0 was considered as the reference group.

### 2.4 Perfusion tissue fixation for immunofluorescence

Animals (five per group) were anaesthetized with cetamina and xylazine (30 and 10 mg/kg i.p). Once the animal was unresponsive to toe‐pinch response (anaesthesia validation test), it was placed on the operating table with its back down. A scalpel was used to make an incision through its abdomen at the length of the diaphragm, followed by a cut of the rib cage up to the collarbone on both sides of the ribs providing a clear view of the heart. A small incision was made in the posterior end of the left ventricle, and an olive‐tipped perfusion needle was inserted through the ventricle to extend straight up about 5 mm. An incision to the rat's right atrium was made to create an outlet for free flow of the solution. A haemostat was used to stabilize the needle and to clamp the descendent aorta to optimize perfusion in the CNS. Five animals per group were perfused with 0.9% sterile saline for 10 minutes (flow rate 20 mL/min) followed by 10 minutes with 4% paraformaldehyde (PFA) solution in PBS (pH 7.4) (flow rate 20 mL/min). The brains were then carefully extracted and maintained in 4% PFA for 24 hours at 4°C, placed in 15% sucrose for 24 hours at 4°C and then placed in 30% sucrose for 24 hours at 4°C. Brains were slightly dried and frozen at −20°C. After 24 hours, the prefrontal cortex and the hippocampus were sectioned in slices of 15 μm on the coronal plane using a cryostat at −20°C (Jung Histoslide 2000R; Leica). A total of 20‐30 slices per rat, containing the structures, were collected in PBS containing 0.1% Triton X‐100 (PBS‐0.1%). The free‐floating sections were incubated with 5% albumin for 2 hours to block nonspecific binding sites.

The antibodies were incubated for 48 hours at 4°C. Anti‐Iba‐1, anti‐CD11b and anti‐arginase were used at 1:500 dilution. DAPI was used for nucleic acid staining (1:500; D9542, Sigma‐Aldrich). Antibodies were diluted in PBS containing 2% bovine serum albumin. After washing four times with 0.1% PBS, tissue sections were incubated with secondary antibodies according to the reactive species (anti‐rabbit or mouse Alexa 488 or 555 from Cell Signaling Technology^®^), all diluted 1:500 in PBS and 2% BSA. After 1 hour at room temperature, the slices were washed several times in 0.1% PBS, transferred to gelatinized slides, mounted with FluorSave™ (345789—Merck Millipore) and covered with coverslips. The images were obtained with a Microscopy EVOS^®^ FL Auto Imaging System (AMAFD1000—Thermo Fisher Scientific). Quantitative analysis of immunofluorescence staining was performed with ImageJ software (ImageJ v1.49, National Institute of Health). Results are expressed in folds relative to the control group.

### 2.5 Analysis of gene expression by real‐time RT‐PCR (RT‐qPCR)

Total RNA was isolated with Trizol^®^ reagent (Invitrogen) in accordance with the manufacturer's instructions. The total RNA was quantified by spectrophotometry (A260/280 nm) after treatment with deoxyribonuclease I (invitrogen) to eliminate genomic DNA contamination in accordance with the manufacturer's instructions. The cDNA was synthesized with ImProm‐II™ Reverse Transcription System (Promega) from 1 µg total RNA, following the manufacturer's instruction. Quantitative PCR was performed using SYBR^®^ Green I (Invitrogen) to detect double‐stranded cDNA synthesis. Reactions were performed in 25 µL volumes using 12.5 µL of diluted cDNA, containing a final concentration of 0.2× SYBR^®^ Green I (Invitrogen), 100 µmol/L dNTP, 1× PCR Buffer, 3 mmol/L MgCl_2_, 0.25 U Platinum^®^ Taq DNA Polymerase (Invitrogen), and 200 nmol/L of each reverse and forward primers classically used as M1 and M2 phenotypes (Table S1). PCR cycling conditions were as follows: an initial polymerase activation step for 5 minutes at 95°C, 40 cycles of 15 seconds at 95°C for denaturation, 35 seconds at 60°C for annealing and 15 seconds at 72°C for elongation. At the end of the cycling protocol, a melting curve analysis was included and fluorescence was measured from 60 to 99°C, all cases showing a single peak. GAPDH was used as reference genes for normalization. Relative expression levels were determined with 7500 Fast Real‐Time System Sequence Detection Software v.2.0.5 (Applied Biosystems). Relative mRNA expression levels were determined using the target/GAPDH method. (Primers are listed in Table S1).

### 2.6 Cytokines levels

Concentrations of IL‐1β, IL‐6, TNF‐α, IL‐10, and CCL‐22 in serum and hippocampus were determined using a commercially available sandwich ELISA kit (R&D Systems) in a microplate reader according to manufacturer's instructions.

### 2.7 Nitrite/Nitrate concentration

Nitrite/nitrate concentration was assayed spectrophotometrically in serum and hippocampus using Griess reagents (1% sulphanilamide in 5% phosphoric acid and 0.1% N‐1‐naphthylethylenediamine dihydrochloride in bi‐distilled H_2_O [NED solution]) and vanadium (III) chloride as previously described.^20^ A standard curve was run simultaneously with each set of samples, and the optical density at 550 nm (OD_550_) was measured using an ELISA microplate reader.

### 2.8 Statistics

Data were presented as mean and standard deviation. Molecular data were analysed with the ExpressionSuite Software v.1.1, expressed as target/GAPDH and percentage change in relation to time 0 (immediately after CLP surgery), and analysed by two‐way analysis of variance (ANOVA), followed by Tukey's post hoc test. All tests were analysed with SPSS version 20 or GraphPad Prism 4.0. In all comparisons, *P* < .05 indicated statistical significance. For immunohistochemistry, immunopositive area was expressed as per cent of total area analysed.

## 3 RESULTS

### 3.1 Hippocampal expression of M1 markers

**Figure 1:**
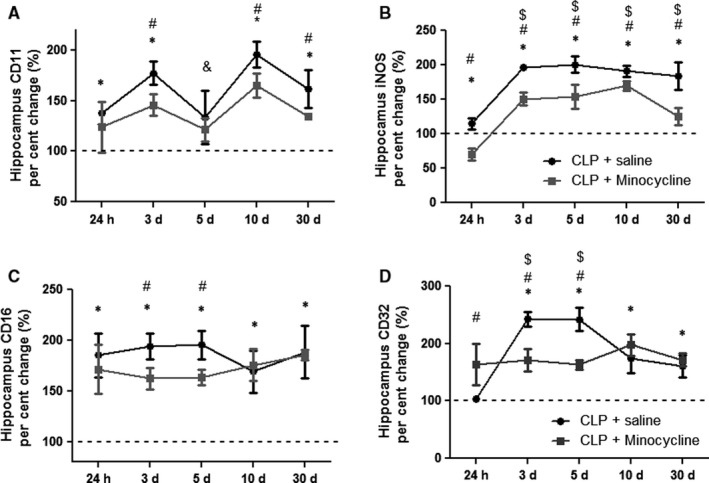
Hippocampal pattern of M1 marker gene expression. M1 markers were measured 24 h, 3, 5, 10 and 30 d after sepsis induction in the hippocampus of animals treated with or without minocycline. (A) CD11; (B) iNOS; (C) CD16; (D) CD32. * different from that at 0 h (dotted line); # different from that of CLP + minocycline at the same time; & different from that at 3‐day (CLP + saline); $ different from that at 24 h (CLP + saline). Data are expressed as mean ± SD *P* < .05

Figure 1 shows the kinetics of M1 markers in the hippocampus of animals subjected to sepsis, with or without minocycline treatment. There was a significant increase in CD11 expression from 24 hours to 30 days, except at 5 days, when compared to expression levels at time 0 in septic animals (Figure 1A). At 5 days, there was a small, but statistically significant, decrease in the expression of CD11 when compared to day 3. Generally, minocycline decreased CD11 expression, reaching statistical significance at 3, 10 and 30 days after CLP induction. iNOS expression was consistently increased early after CLP, and it kept increasing until day 30 (Figure 1B). The effect of minocycline was down‐regulation of iNOS expression across all studied times. Similar to iNOS, there was a consistent increase in CD16 levels early after sepsis induction that was maintained until 30 days after CLP induction (Figure 1C). Minocycline induced significant inhibition of CD16 expression during the first 5 days after sepsis induction, but had no effects from days 10 to 30. The pattern of CD32 expression was somewhat different when compared to that of other M1 markers (Figure 1D). There was a late (3 and 5 days), but sustained increase in its expression. Minocycline abrogated up‐regulation of CD32 expression after sepsis.

### 3.2 Hippocampal expression of M2 markers

**Figure 2:**
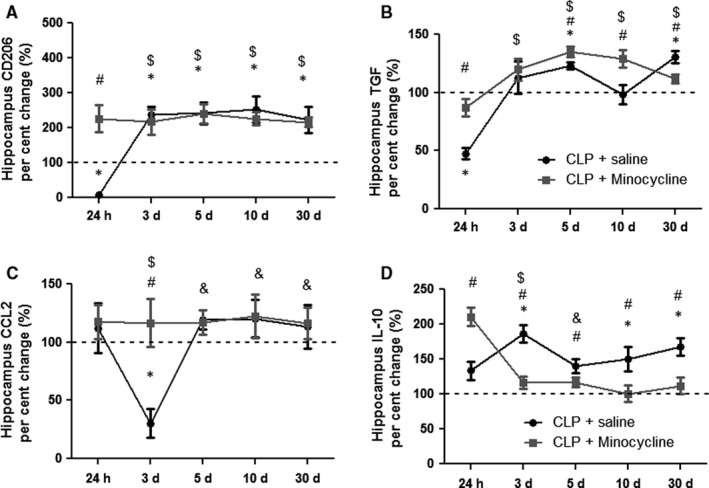
Hippocampal pattern of M2 markers gene expression. M2 markers were measured 24 h, 3, 5, 10 and 30 d after sepsis induction in the hippocampus of animals treated with or without minocycline. CD206 (A); TGF (B); CCL‐22 (C); IL‐10 (D). * different from that at 0 h (dotted line); # different from that of CLP + minocycline at the same time; & different from that at 3 d (CLP + saline); $ different from that at 24 h (CLP + saline). Data are expressed as mean ± SD *P* < .05

Figure 2 shows the kinetics of M2 markers in the hippocampus of animals subjected to sepsis, with or without minocycline treatment. CD206 expression significantly decreased early after sepsis induction, followed by significant increase after 3 days, which was maintained until day 30 (Figure 2A). Interestingly, minocycline transitorily increased CD206 expression after 24 hours, but did not interfere in CD206 expression at any other time (Figure 2A). TGF had an expression pattern similar to CD206; there was an early decrease followed by a late increase in its expression from days 5 to 30 after CLP, and minocycline transiently increased its expression after 24 hours, and 5 and 10 days when compared to CLP (Figure 2B). However, 30 days after sepsis induction, CLP animals had higher levels of TGF when compared to minocycline‐treated animals (Figure 2B). Figure 2C shows CCL‐22 expression after CLP. There was a transient, but significant decrease 3 days after CLP. Minocycline treatment prevented the decrease of CCL‐22 expression at day 3, but in general, there was no regulation of this marker after sepsis. In addition, IL‐10 significantly increased 3 days after CLP, with these levels being maintained until day 30. Again, minocycline induced an early increase in IL‐10 expression at 24 hours, and from day 3 to 30, decreased its expression after CLP.

### 3.3 Hippocampal levels of M1 cytokines and nitrite/nitrate

**Figure 3:**
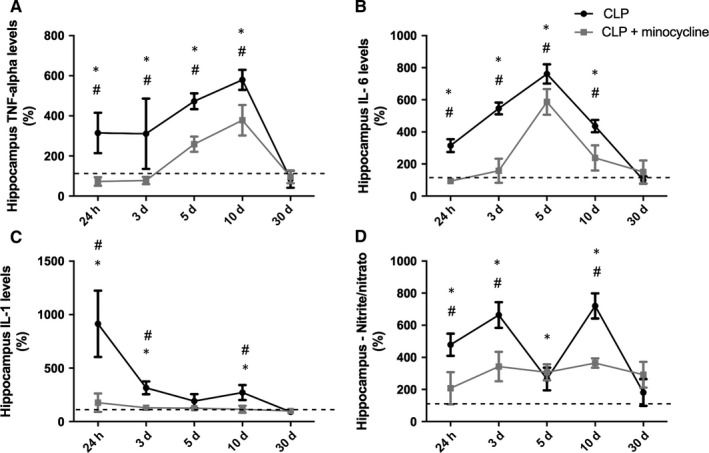
Hippocampal pattern of M1 cytokines/ nitrite levels. M1 cytokines levels were measured 24 h, 3, 5, 10 and 30 d after sepsis induction in the hippocampus of animals treated with or without minocycline. (A) TNF; (B) IL‐6; (C) IL‐1; (D) nitrite/nitrate. * different from that at 0 h (dotted line); # different from CLP + minocycline at the same time. Data are expressed as mean ± SD *P* < .05

Figure 3 shows the kinetics of M1 cytokines in the hippocampus of animals subjected to sepsis, treated or not with minocycline. TNF‐α (A), IL‐6 (B) and IL‐1 (C) levels remained increased until 10 days after sepsis, decreasing to basal levels by day 30. Minocycline was able to decrease these levels at all analysed times. Nitrite/nitrate levels (D) were significantly increased until day 10 when compared to time 0. Minocycline reduced these levels at all times except on day 5.

### 3.4 Hippocampal levels of M2 cytokines

**Figure 4:**
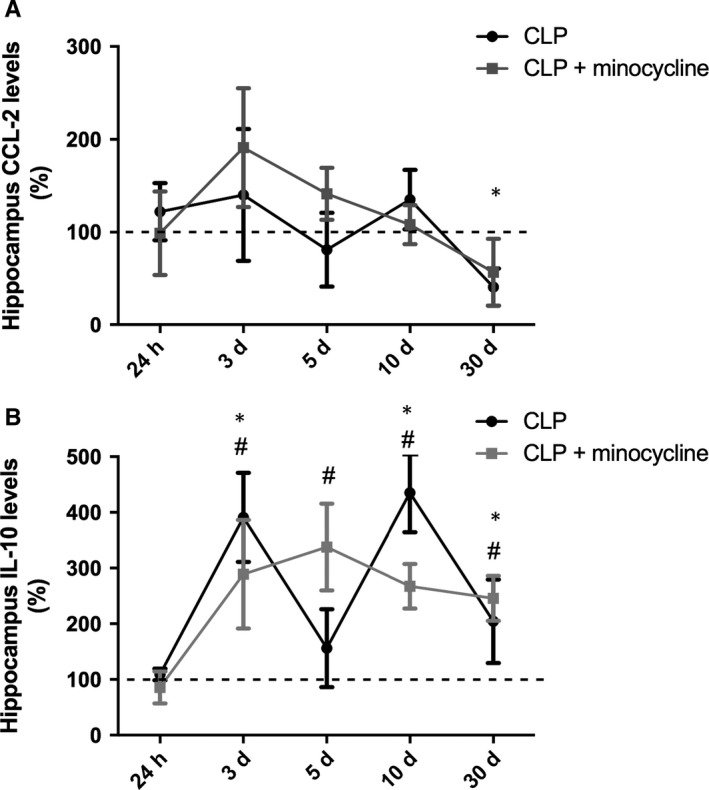
Hippocampal pattern of M2 cytokines levels. M2 cytokines levels were measured 24 h, 3, 5, 10 and 30 d after sepsis induction in the hippocampus of animals treated with or without minocycline. (A) CCL‐22; (B) IL‐10. * different from that at 0 h (dotted line); # different from CLP + minocycline at the same time. Data are expressed as mean ± SD *P* < .05

There was no alteration in CCL‐22 levels until day 30 (Figure 4A). IL‐10 levels peaked at days 3 and 10 and remained elevated for up to 30 days (Figure 4B). Minocycline increased IL‐10 levels 5 days after sepsis (Figure 4B).

### 3.5 Hippocampal immunofluorescence of CD11b (M1 marker)

**Figure 5:**
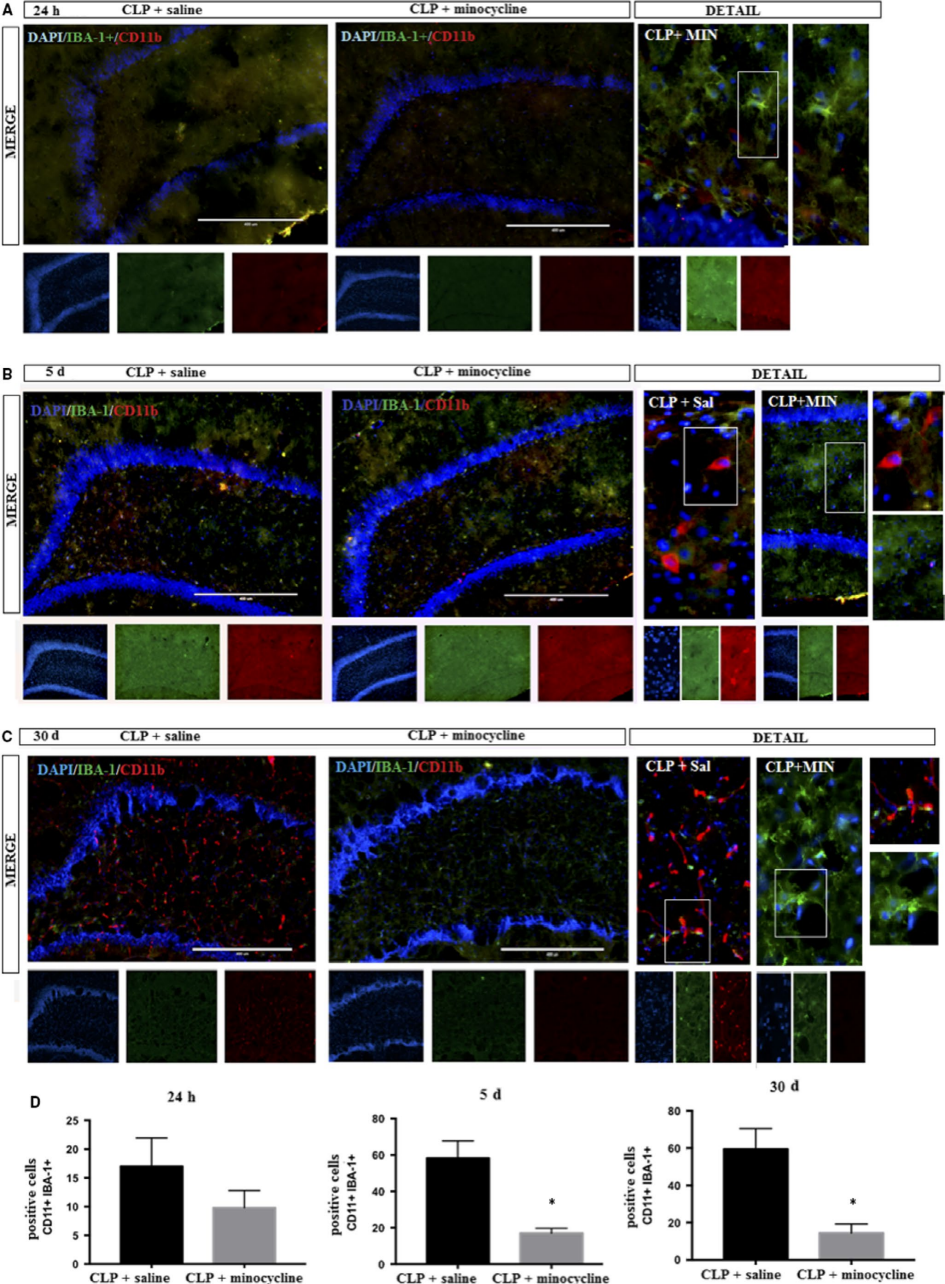
DAPI (nuclei‐ blue); CD11b (M1 marker– red); IBA‐1+ (microglial marker—green) was determined by immunofluorescence, 24 h, 5 and 30 d after sepsis in the hippocampus of animals treated with or without minocycline. A, 24 h; B, 5 d; C, 30 d; D, corresponding graphs. Image details are presented. Double positive cells (CD11b + IBA‐1+) were counted in both groups. T‐test for independent samples. *P* = .18; *P* = .03

Figure 5 shows the kinetics of CD11b (M1 marker) by immunofluorescence. There was a progressive increase in CD11 + cells for 5 days until 30 days after CLP. From day 5 to day 30, minocycline decreased the number of CD11+ cells.

### 3.6 Hippocampal immunofluorescence of arginase (M2 marker)

**Figure 6:**
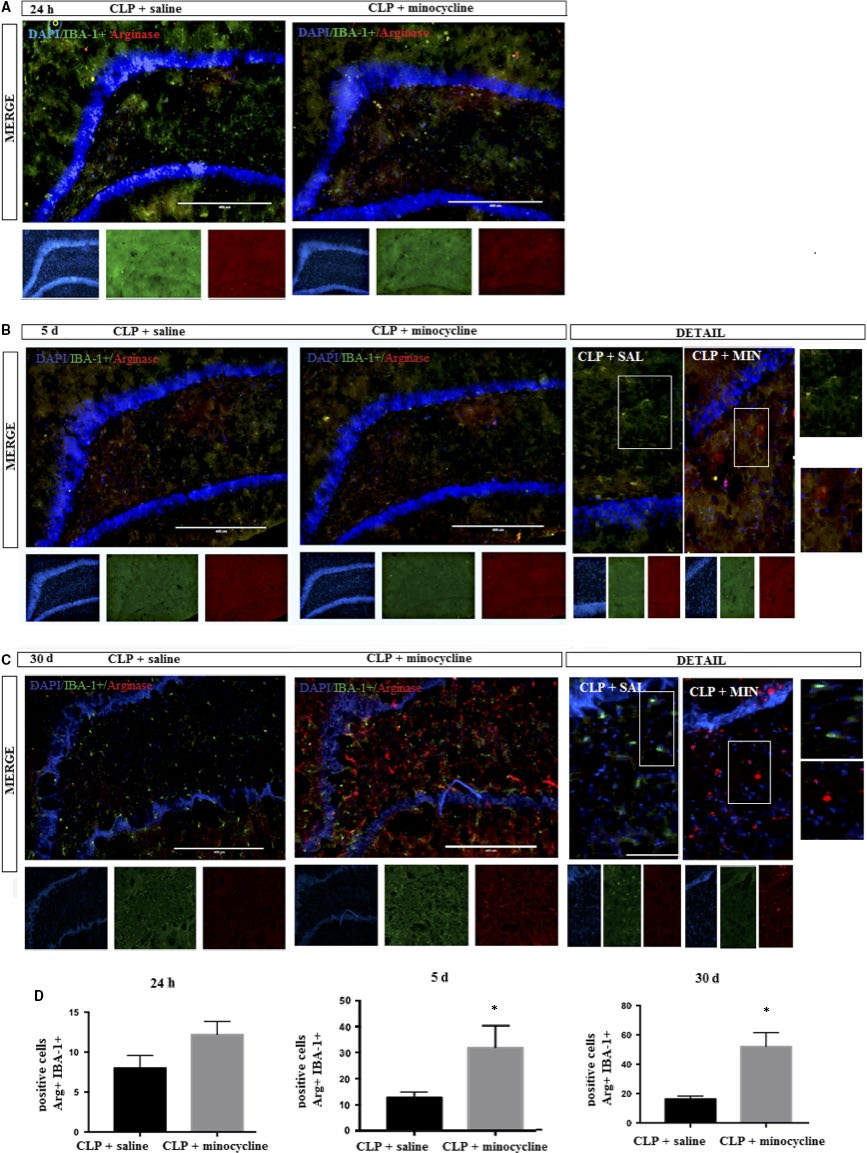
DAPI (nuclei—blue); arginase (M2 marker—red); IBA‐1+ (microglial marker—green) was determined by immunofluorescence, 24 h, 5 and 30 d after sepsis in the hippocampus of animals treated with or without minocycline. A, 24 h; B, 5 d; C, 30 days; D, corresponding graphs. Image details are presented. Double positive cells (CD11b + IBA‐1+) were counted in both groups. *t* Test for independent samples. *P* = .74; *P* = .03

Minocycline treatment resulted in the early appearance of arginase+ cells (from 5 days after CLP), and the number of positive cells in this group was higher when compared to that after CLP at 30 days (Figure 6).

### 3.7 Serum levels of M1/M2 cytokines and nitrite/nitrate

**Figure 7:**
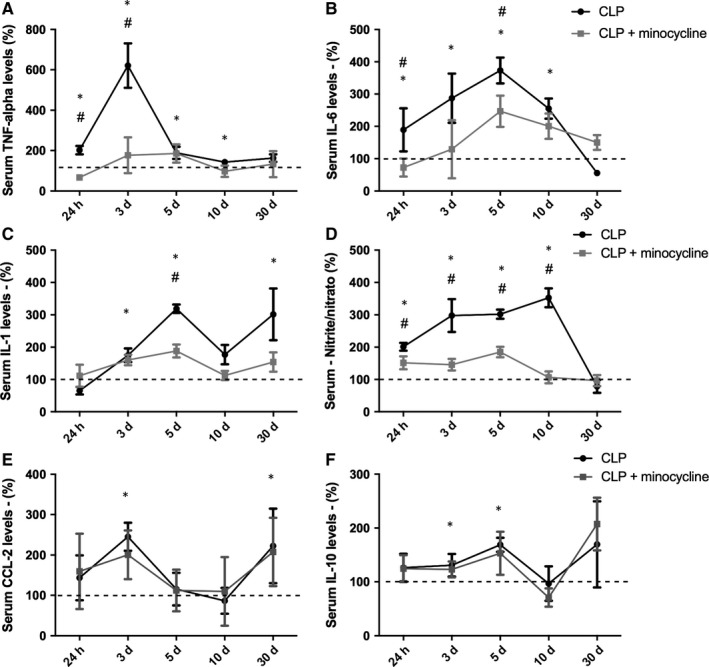
Serum pattern of M1/M2 cytokines and nitrite levels. Cytokines and nitrite/nitrate levels were measured 24 h, 3, 5, 10 and 30 d after sepsis induction in the hippocampus of animals treated with or without minocycline. A, TNF; B, IL‐6; C, IL‐1; D, nitrite/nitrate; E, CCL‐22; F, IL‐10. *Different from that at 0 h (dotted line); # different from CLP + minocycline at the same time. Data are expressed as mean ± SD *P* < .05

To determine the effect of i.c.v. administration of minocycline on systemic inflammatory response, levels of M1/M2 cytokines and nitrite/nitrate were determined in the serum of animals subjected to CLP treated with or without minocycline. As expected, TNF‐α, IL‐6 and IL‐1β increased early after CLP induction and peaked from 24 hours to 5 days after (Figure 7 A‐C). These M1 cytokines remained slightly, but significantly, increased until 10 to 30 days after sepsis. Minocycline attenuated the magnitude of the M1 cytokine peak and the duration of this increase (Figure 7A‐C). The same behaviour was observed for serum nitrite/nitrate levels (Figure 7D). Conversely, CCL‐22 and IL‐10 increased late after sepsis (from day 3 to day 30), but i.c.v. minocycline did not modulate IL‐10 and CCL‐22 serum levels (Figure 7E‐F).

## 4 DISCUSSION

Here, we demonstrated, using gene expression, cytokine levels and surface markers, that early after sepsis, there is a preponderant up‐regulation of M1 microglial markers, and as we hypothesized, this pattern did not later switch to the M2 phenotype. We have demonstrated that up‐regulation of both M1 and M2 markers co‐exists up to 30 days after sepsis induction. In addition, minocycline induced a down‐regulation, predominantly, of M1 markers, suggesting that its protective effects after sepsis are due to, at least in part, the induction of a predominant M2 phenotype.

The multiple activation of phenotypes in microglial cells is a relatively new perception and is mainly derived from macrophage literature. In this context, specifically in the sepsis field, there are few reports about the behaviour of different microglial phenotypes. For instance, D'Avila et al^21^ demonstrated that aged mice produced higher levels of pro‐inflammatory cytokines (IL‐1β and IL‐6) as well as pro‐resolution (IL‐10 and IL‐4) in the brain after episodic systemic inflammation when compared to young animals. Additionally, aged mice presented dystrophic microglia at basal level and did not change morphology in the response to systemic inflammation.^21^ We recently demonstrated that the depletion of microglia before sepsis development is associated with acute exacerbation of brain inflammation. Furthermore, microglia repopulation is able to revert this, mainly inducing a shift to M2 phenotype in these animals.^22^ Additionally, in septic humans, it was found pro‐inflammatory microglia, and the most important difference between patients with sepsis and controls was the induction of iNOS in microglia. Interestingly, the expression of the P2RY12 (homeostatic microglia marker) was similar in control and septic brains.^23^

Based on macrophage literature, it is classically expected that within 24 hours after insult, monocytes are recruited from circulation, and they traffic into an infection site, where they mature into classically activated macrophages, also called M1 macrophages.^24^, ^25^ Later, macrophages usually switch to M2 phenotypes that are involved in inflammation resolution.^24^, ^25^ Whether proliferating microglia might migrate and settle in specific brain areas, and the phenotype associated with acute and late phases of sepsis where they are possibly involved in re‐establishing tissue homeostasis, is not known.

We observed that all M1 markers were initially up‐regulated in comparison to CLP time 0, and this was even more apparent when compared to the kinetics of M2 markers. Interestingly, minocycline seemed to act more robustly on M1 markers. The activation of M1 without counterbalancing M2 could be important to the brain's acute response to systemic inflammation, but may present negative results in the long‐term, since minocycline improved long‐term cognitive deficits in sepsis survivor.^3^ Minocycline seems to induce a more balanced response, mainly at early times, decreasing brain M1 markers and attenuating a systemic inflammatory response. Studies have shown that minocycline reduces the levels of pro‐inflammatory cytokines,^2^ and here we demonstrated that it acts on different aspects of microglia activation (gene expression, membrane markers and cytokine secretion). Minocycline was also able to significantly decrease iNOS expression and nitrite/nitrate levels, which suggests an indirect protective function of minocycline by decreasing free radical production,^24^, ^26^ which was previously thought to be associated with long‐term cognitive impairment after sepsis.^27^

M2 macrophages, which demonstration anti‐inflammatory properties, are classically defined by the expression of the scavenger (CD163) and mannose (CD204/206) receptors as well as enhanced expression and secretion of immunosuppressive cytokines, such as IL‐10 and transforming growth factor (TGF)‐β1.^28^ In addition, the expression of arginase 1 (Arg1) counteracts the effects of iNOS from M1 macrophages.^25^ The kinetics of M2 during sepsis development seems to be a regulatory response to counterbalance M1 activation. However, M2 markers appear late after sepsis induction and the magnitude of their up‐regulation is generally less robust when compared to M1 markers. This is further reinforced by the fact that the M1 cytokines, IL1, IL‐6 and TNF are consistently increased in the hippocampus late after sepsis, and IL‐10 has some peaks during sepsis evolution and CCL‐22 is not increased at all. In this context, minocycline seems to decrease M1 markers and simultaneously increase M2, resulting in a more balanced phenotype expression. Zhao et al^29^ showed that minocycline suppressed the production of pro‐inflammatory molecules, reduced Iba1 (+) cells and reversed the reduction of M2 microglial markers in animal models of maternal sleep deprivation.

One interesting finding is the fact that the modulation of brain inflammation seems to regulate systemic inflammatory response. The i.c.v. administration of minocycline decreased the magnitude of serum M1 response, and this mirrored, to some extent, the in situ effect of minocycline. This suggests a crosstalk between microglia and macrophage phenotypes that could be driven by brain cytokine patterns. It is possible that the magnitude of brain inflammation modulates some aspects of brain‐systemic inflammatory response, such as the integrity of the blood‐brain barrier, activation of endothelial cells, and activation of the anti‐cholinergic or hypothalamic‐pituitary‐adrenal axes.^30^, ^31^ For example, it was demonstrated that minocycline selectively protects cholinergic neurons from p75‐saporin toxicity 32 and there is a crosstalk between hippocampal and splenic inflammation in knockout mice for the vesicular acetylcholine transporter.^33^

One relevant point that was not addressed by our study is whether minocycline administration improves mortality in this model. Despite it was not the main objective of this study, this is a clinically and scientifically relevant question. It is known that systemic administration of minocycline improves mortality in CLP model,^34^ but this could be secondary to its antibiotic effect. When administered directly in the brain, minocycline improved brain inflammation and dysfunction,^3^, ^35^ and this could impact in survival. Further studies are necessary to specifically answer this.

Some limitations must be highlighted. First, gene expression analysis was not performed in isolated microglia from septic rats; thus, because neurons, glia and endothelial cells also express some of the analysed markers, we cannot ascertain whether our results reflect only the microglia expression of these markers. This limitation is minimized by the fact that microglia phenotypes were determined by three different methods. Second, blood‐borne leucocytes can also reach the brain during sepsis development, and they probably share the same phenotypes and are inhibited by minocycline 36; thus, some of the observed effects could be associated with blood‐borne leucocytes. Third, despite being highly improbable that low doses of i.c.v. administered could directly inhibit blood‐borne leucocytes, we cannot ascertain whether the effects of minocycline on systemic inflammation are only secondary to decreased brain inflammation.

## 5 CONCLUSION

In conclusion, early activation of M1 microglia is followed by an overlap of both M1 and M2 phenotypes. The beneficial effects of minocycline on sepsis‐associated brain dysfunction may be related to its effects predominantly on the M1 phenotype. These findings might reflect the importance of the M1/M2 balance in brain repair after systemic inflammation.

## CONFLICT OF INTEREST

The authors declare no competing financial interests.

## AUTHOR CONTRIBUTIONS

MM contributed to experimental planning, data collection, data analysis, and writing of paper; MRA, PA, AV contributed to experimental planning, and data analysis; HB, CCJ, DW, JG, CTR contributed to data analysis; JCFM, DPG and FDP contributed to experimental planning and writing of paper.

## Data Availability

The data that support the findings of this study are available from the corresponding author FDP upon reasonable request.
