# Supplementary material for: Characterization and modulation of microglial phenotypes in an animal model of severe sepsis
Source: J Cell Mol Med. 2019 Oct 26;24(1):88–97. doi: 10.1111/jcmm.14606 (PMC6933367; doi:10.1111/jcmm.14606)
Supplement: Supplementary file 1 [file JCMM-24-88-s001.docx]

| **iNOS** | SENS: CAAGCACCTTGGAAGAGGAG  REVS: AAGGCCAAACACAGCATACC; |
| --- | --- |
| **CD32** | SENS: AATCCTGCCGTTCCTACTGATC  REVS: GTGTCACCGTGTCTTCCTTGAG; |
| **CD16** | SENS: TTTGGACACCCAGATGTTTCAG  REVS: GTCTTCCTTGAGCACCTGGATC; |
| **CD86** | SENS: GACCGTTGTGTGTGTTCTGG  REVS: GATGAGCAGCATCACAAGGA; |
| **CD11b** | SENS: CCAAGACGATCTCAGCATCA  REVS: TTCTGGCTTGCTGAATCCTT; |
| **CD206** | SENS:CAAGGAAGGTTGGCATTTGT  REVS: CCTTTCAGTCCTTTGCAAGC; |
| **Arg1** | SENS: TCACCTGAGCTTTGATGTCG  REVS: CTGAAAGGAGCCCTGTCTTG; |
| **IL-10** | SENS: CCAAGCCTTATCGGAAATGA  REVS: TTTTCACAGGGGAGAAATCG; |
| **CCL-22** | SENS: CTGATGCAGGTCCCTATGGT  REVS: GCAGGATTTTGAGGTCCAGA; |
| **TGF-β** | SENS: TGCGCTTGCAGAGATTAAAA  REVS: CGTCAAAAGACAGCCACTCA; |
| **Ym1/2** | SENS:CAGGGTAATGAGTGGGTTGG  REVS: CACGGCACCTCCTAAATTGT |
| **GAPDH** | SENS: AATGGGGTGATGCTGGTGCTGA  REVS: TGGGGGCTGAGTTGGGATGG |
